# Estimating the probability of dengue virus introduction and secondary autochthonous cases in Europe

**DOI:** 10.1038/s41598-018-22590-5

**Published:** 2018-03-15

**Authors:** Eduardo Massad, Marcos Amaku, Francisco Antonio Bezerra Coutinho, Claudio José Struchiner, Marcelo Nascimento Burattini, Kamran Khan, Jing Liu-Helmersson, Joacim Rocklöv, Moritz U. G. Kraemer, Annelies Wilder-Smith

**Affiliations:** 1 School of Medicine, University of Sao Paulo, Sao Paulo, Brazil; 2 London School of Hygiene and Tropical Medicine, London, UK; 3 College of Natural and Life Sciences, The University of Derby, Derby, UK; 4 501100007227 School of Applied Mathematics, Fundação Getúlio Vargas, Rio de Janeiro, Brazil; 5 Programme of Scientific Computation, Fundação Oswaldo Cruz, Rio de Janeiro, Brazil; 6 Hospital São Paulo, Escola Paulista de Medicina, Universidade Federal de São Paulo, São Paulo, SP Brazil; 7 Li Ka Shing Knowledge Institute, St Michael’s Hospital, Toronto, Canada; 8 Department Public Health and Clinical Medicine, Epidemiology and Global Health, Umea University, SE-901 85 Umea, Sweden; 9 Harvard Medical School & Boston Children’s Hospital & Department of Zoology, University of Oxford, Oxford, UK; 10 Institute of Public Health, University of Heidelberg, Heidelberg, Germany; 11 Lee Kong Chian School of Medicine, Nanyang Technological University, Singapore, Singapore

## Abstract

Given the speed of air travel, diseases even with a short viremia such as dengue can be easily exported to dengue naïve areas within 24 hours. We set out to estimate the risk of dengue virus introductions via travelers into Europe and number of secondary autochthonous cases as a result of the introduction. We applied mathematical modeling to estimate the number of dengue-viremic air passengers from 16 dengue-endemic countries to 27 European countries, taking into account the incidence of dengue in the exporting countries, travel volume and the probability of being viremic at the time of travel. Our models estimate a range from zero to 167 air passengers who are dengue-viremic at the time of travel from dengue endemic countries to each of the 27 receiving countries in one year. Germany receives the highest number of imported dengue-viremic air passengers followed by France and the United Kingdom. Our findings estimate 10 autochthonous secondary asymptomatic and symptomatic dengue infections, caused by the expected 124 infected travelers who arrived in Italy in 2012. The risk of onward transmission in Europe is reassuringly low, except where Aedes aegypti is present.

## Introduction

**Figure 1:**
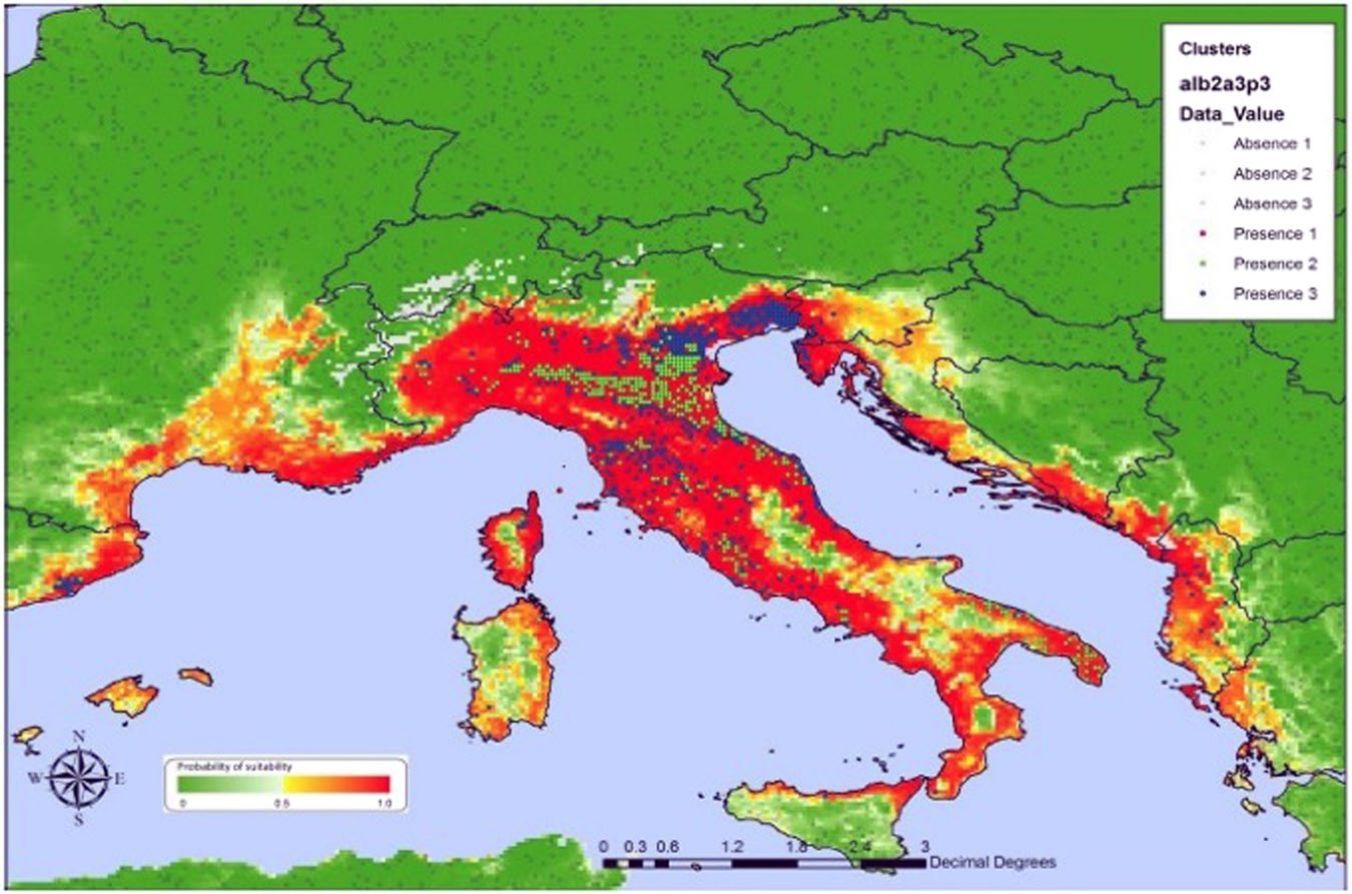
European countries in which *Aedes albopictus* was present in 2012 (From Environmental Risk Mapping: *Aedes albopictus*in Europe. European Centre for Disease Prevention and Control (ECDC Technical Report), Environmental risk mapping: Aedes albopictus in Europe, Stockholm: ECDC; 2013, available at: https://www.google.com.br/url?sa=t&rct=j&q=&esrc=s&source=web&cd=2&ved=0ahUKEwjVuMCy1v7UAhVJIJAKHQIxB_kQFggpMAE&url=http%3A%2F%2Fecdc.europa.eu%2Fen%2Fpublications%2FPublications%2Fclimate-change-environmental-risk-mapping-aedes.pdf&usg=AFQjCNEKPAVsU4vqMfGXjL4pHcqy9EXz-A&cad=rja.

Arboviral diseases are on the rise, with dengue viral infections taking the lead^1^. The distribution of *Aedes aegypti* and *Ae*. *albopictus* mosquitoes - the vectors for dengue- is now the widest ever recorded; extensive in all continents, including North America and Europe, with over three billion people living in *Aedes*-infested countries or areas^2^. Although *Aedes aegypti*, remains a vector predominantly of the tropics and subtropics, the global spread of *Ae*. *albopictus* fuelled by global trade and travel^3^, and its resistance to colder weather^2^, put temperate areas such as Europe at risk for transmission of dengue and other *Aedes* transmitted viruses. Figure 1 depicts the distribution of *Aedes albopictus* in Europe. Some model studies predict that climate change will increase dengue epidemic potential in temperate regions, potentially flattening the differences between tropical and temperate zones^4–6^. Compared with the tropics, Europe shows pronounced seasonality which affects temperature-dependent proliferation of *Aedes* mosquitoes. Although vectorial capacity – the mosquito’s capacity to transmit dengue virus to humans – is low in Europe according to modeling estimates, vectorial capacity is sufficient during the summer months for a dengue outbreak to occur in most of Southern Europe^4^. Indeed, small autochthonous dengue clusters occurred in Southern France and in Croatia in 2010^7^, and a major dengue outbreak involving more than 2000 persons occurred in Madeira/Portugal in 2012^8^, underpinning that the threat of dengue to Europe is real. The potential for dengue outbreaks to occur in Europe depends on two main factors: (1) the presence of *Aedes* vectors during the summer season when vectorial capacity is sufficient to sustain transmission and (2) the rate of dengue virus importations, that depends on the number of dengue viremic travelers entering Europe. Most of the modeling work has been focused on understanding the risk of local transmission and relatively little attention has been given to the extent of disease importations. As human movements resulting in global spread of infectious diseases including vector-borne diseases are increasing^9–18^ and the burden of diseases such as dengue increases, we can expect an exponential increase in arrivals of viremic passengers which may challenge traditional health infrastructures.

Given the speed of air travel today^19^ and increase of travel to tropical and subtropical countries^19^, diseases with short viremia such as dengue (around 7 days) can be easily exported to a dengue naïve areas within 24 hours, where mosquitoes may then be able to feed on viremic blood, and transmit the virus on to other humans. A sentinel surveillance study recently estimated that about 40% of travelers diagnosed with dengue are viremic at the time of arrival in Europe^20^. The first large dengue outbreak in Europe that occurred in Madeira in 2012 as a result of importation of the virus via incoming viremic air passengers most likely from Venezuela^8^ prompted us to study the extent of potentially dengue viremic travelers arriving in Europe as a whole in the same year and to estimate the probability of secondary transmission in Europe as a result of such importation. In the absence of good empiric data on importation of dengue via viremic travelers, mathematical models can provide an additional tool to estimate the number of dengue virus introductions^21^. The extent of dengue virus introduction is a function of travel volume and dengue incidence in the ‘exporting’ country^22,23^. We set out to model the estimated numbers of dengue-viremic air passengers from dengue-endemic countries to 27 European countries and the subsequent risk of autochthonous transmission as a result of such importation.

## Methods

We applied a previously published mathematical model to estimate the risk of importation of infectious diseases via travelers^21^, and expanded it to further to include models on vectorial capacity^4,5^. To estimate the number of dengue viremic air passengers into Europe in one year we took into account air travel volume, the dengue monthly incidence in the country of origin, and the probability for the air passenger to be viremic at the time of travel.

We consider two types of countries, the ‘exporting’ country (where the infection is endemic) and those ‘importing’ or ‘receiving’ countries. We investigated the number of imported dengue-viremic travelers into 27 European countries based on the following variables: (1) the monthly dengue incidence in the exporting countries; (2) the monthly number of people leaving the airports of exporting countries and traveling to importing countries; (3) the expected monthly number of dengue-viremic travelers arriving at the importing countries. (4) the accumulated number of secondary cases in humans in the importing countries generated by the infected travelers from the exporting countries taking into account the vectorial capacity of Aedes mosquitoes in the importing countries over a one year period; and (5) the accumulated per capita risk of dengue infection in the population of the importing countries over one year caused by infected travelers arriving in that year. Items 4 and 5 will be exemplified by the cases of Italy, in which *Aedes albopictus* is currently present, and Madeira, where *Aedes aegypti* caused an important outbreak in 2012. *Aedesalbopictus* is a much less competent mosquito for dengue transmission than *Aedes aegypti*; Tables S2 and S3 in the supplement list the values of the transmission parameters that we used for our models on vectorial capacity.

### Estimating the incidence of dengue in the exporting countries

The first step was to obtain information on dengue cases notified to the World Health Organization (WHO) from their website (www.who.int). From the 85 countries that reported 1,597,220 dengue cases to WHO in 2012 we selected 16 countries which were responsible for 95% of all the cases. These countries, along with the number and relative contribution to the total number of dengue cases are shown in Table S1 (supplementary material). Of these 16 selected countries, 9 are from South and Central America, and the rest from Asia. As cases are only reported to WHO on an annual basis, we inferred the seasonal distribution per month from those two countries where we had monthly data: Brazil and Thailand. We assumed that the 9 South and Central American countries had seasonality similar to that of Brazil. The remaining 7 Asian countries were assumed to have the same seasonality as Thailand. The seasonal pattern of these two reference countries, Brazil and Thailand, are shown in figures S1 to S4 (supplementary material). We assumed that visitors to the exporting countries were subject to the same risk of infection as the local inhabitants.

### Estimating the monthly number of people leaving the airports of exporting countries and traveling to importing countries

The second step is to fit a continuous function to the number of actually reported dengue cases (incidence) multiplied by 4 to take into account the 4:1 asymptomatic:symptomatic ratio for dengue infections^24^. The continuous function chosen has the form:

1 $$Incidenc{e}_{DENV}(t)={c}_{1}\,\exp [-\frac{{(t-{c}_{2})}^{2}}{{c}_{3}}]+c$$

representing the time-dependent dengue infection incidence. In equation (1) *c*_1_ is a scale parameter that determines the maximum incidence, *c*_2_ is the time at which the maximum incidence is reached, *c*_3_ represents the width of the time-dependent incidence function and *c*_4_ is a time and location dependent parameter to counter the seasonal differences in dengue incidence between Northern and Southern Hemisphere. Equation (1) reproduces a “Gaussian” curve and so *c*_1_ and *c*_4_ are just scale parameters but *c*_2_ crepresents the “mean” (and mode or maximum) time and *c*_3_ represents the “variance” of the time distribution of cases. All parameters *c*_1_,*i* = 1, …, 4 fitted to function (1), when used in the dynamical model described below reproduces the observed incidence of dengue for a given outbreak in a region preferably small as we will explain later. Parameter *c*_4_ is given by:

2 $${c}_{4}=\{\begin{array}{c}rect(t),\,{\rm{for}}\,{\rm{Southern}}\,{\rm{Countries}}\\ 0,{\rm{for}}\,{\rm{Northern}}\,{\rm{Countries}}\end{array}\,$$

where *rect* (*t*) is a rectangular function added to equation (1) to take account of the slight increase in the risk at the end of the year in the Southern Countries (summer time). The rectangular function is a square pulse of determined duration and can be written in terms of the Heaviside step function as *θ*(*t* + *t*_*i*_) − *θ*(*t* − *t*_*i*_). The *rect* (*t*) is zero for any *t* > |*t*_*i*_| and is equal to 1 for *t* < |*t*_*i*_|.

The number of monthly reported dengue cases of all 16exporting countries, was used to estimate the monthly prevalence of dengue in each country. Therefore, all the quantities should have a superscript *j* (*j* = *1 … 12)* denoting the months of the year. We used previously developed and validated models (1) and (2)^21,24–26^ to the human components of the Ross-Macdonald model, in which ${S}_{H}^{j}(t)$, ${I}_{H}^{j}(t)$ and ${R}_{H}^{j}(t)$ represent the susceptible, infected and recovered humans in each month of the year, respectively and *μ*_*H*_ and *γ*_*H*_ are the humans natural mortality and recovery from infection rates, respectively.

The model is described by the following set of equations:

3 $$\begin{array}{rcl}\frac{d{S}_{H}^{j}}{dt} & = & -Incidenc{e}_{DENV}^{j}(t)+{\mu }_{H}({I}_{H}^{j}+{R}_{H}^{J})\\ \frac{d{I}_{H}^{j}}{dt} & = & Incidenc{e}_{DENV}^{j}(t)+({\mu }_{H}+{\gamma }_{H}){I}_{H}^{j}\\ \frac{d{R}_{H}^{j}}{dt} & = & {\gamma }_{H}{I}_{H}^{j}-{\mu }_{H}{R}_{H}^{J}\\ {N}_{H}^{j}(t) & = & {S}_{H}^{j}(t)+{I}_{H}^{j}(t)+{R}_{H}^{j}(t)\\ {\rm{for}}\,t &  >  & {t}^{j}\end{array}$$

such that the parameters *c*_*i*_, *i* = *1*, *…*, *4* of equation (1) reproduce the incidence data (reported cases). This system is valid for any specific dengue serotype. As we are working with reporting cases of dengue, we are not concerned with the particular serotype circulating.

The first term of the first equation in system of equations (3) ($Incidenc{e}_{DENV}^{j}(t)$) models the number of new infections per time unit. In terms of the classical notation of vector-borne infections^27^ it is equal to the product of the force-of-infection, *λ*^*j*^(*t*) times the number of susceptible humans, denoted ${S}_{H}^{j}(t)$. As is well known, the force-of-infection in vector-borne infections is the product of the biting rate times the probability of transmission from infected mosquitoes to the human hosts, times the number of infected mosquitoes divided by the total number of humans^27^.

*Remark: λ*^*j*^(*t*)${S}_{H}^{j}(t)$ *is the dengue incidence:*

$$Incidenc{e}_{DENV}^{j}(t)={\lambda }^{j}(t){S}_{H}^{j}(t)=ab\frac{{I}_{M}^{j}(t)}{{N}_{H}^{j}}{S}_{H}^{j}(t).$$

The individual probability of being infected at time *t*, defined as the monthly individual risk of being infected and denoted $Ris{k}_{DENV}^{j}(t)$, which is given by the prevalence of dengue:

4 $$Ris{k}_{DENV}^{j}(t)=\frac{{I}_{H}^{j}(t)}{{N}_{H}^{j}},\,{\rm{for}}\,t > {t}^{j}$$

This risk is obtained as follows. From the second equation of model (3) for the infected humans we obtain:

5 $$\frac{d{I}_{H}^{j}(t)}{dt}=Incidenc{e}_{DENV}^{j}(t)-({\mu }_{H}+{\gamma }_{H}){I}_{H}^{j}(t),$$

which can be integrated by standard methods to obtain:

6 $${I}_{H}^{j}(t)={I}_{H}^{j}(0){e}^{-({\mu }_{H}+{\gamma }_{H})t}+{\int }_{{t}^{j}}^{t}{e}^{({\mu }_{H}+{\gamma }_{H})(x-t)}Incidenc{e}_{DENV}^{j}(x)dx.$$

Note that the concept of risk expressed in equation (4) means the probability of finding at least one dengue case at month *j*, either among travellers who visited each of the selected countries or among local inhabitants. In addition, equation (4) includes the notion that a proportion of the infected individuals can recover from the infection or die after they arrive at one of the European countries.

### Estimating the number of dengue-viremic air passengers arriving in the importing countries

We obtained the expected number of passengers arriving in European countries infected with dengue in the exporting countries by multiplying equation (4) by the number of air passengers from the 16 selected countries with final destinations in any of the 27 European countries. We analysed worldwide full-route flight itinerary data, taking into consideration all flight connections between those 16 exporting countries and 27 receiving European countries, from the International Air Transport Association (IATA) between 1 January and 31 December 2012.

### Estimating the monthly number of secondary dengue infections in humans in the importing countries generated by dengue-viremic travelers from the exporting countries based on vectorial capacity in that country

In order to estimate the monthly number of autochthonous mosquitoes and humans we used again the Ross-Macdonald in its full version.

Infected (i.e., dengue-viremic) travellers arrive at each month *j* of the year and are denoted ${I}_{H}^{j}(t)=$ ${I}_{H}^{j}({t}^{j}){e}^{-({\mu }_{H}+{\gamma }_{H})(t-{t}^{j})}\theta (t-{t}^{j})$. In the model we denote the mosquitoes densities as ${m}^{j}(t)\frac{{I}_{M}^{I}(t)}{{N}_{H}^{I}}$ and the superscript *I* denotes importing countries. The mosquitoes densities ${S}_{M}^{I}$, ${L}_{M}^{I}$ and ${I}_{M}^{I}$, representing the susceptible, latent and infected mosquitoes, respectively, were calculated according to the methods described in.^4^ Total human population sizes, ${N}_{H}^{I}$, are obtained from demographic data for each country. The model has the form:

7 $$\begin{array}{rcl}\frac{d{S}_{H}^{I}}{dt} & = & -{S}_{H}^{I}(t)\sum _{j=1}^{12}{a}^{j}(t){b}^{j}(t){m}^{j}(t)+{\mu }_{H}[{I}_{H}^{I}(t)+{R}_{H}^{I}(t)]\\ \frac{d{S}_{H}^{I}}{dt} & = & {S}_{H}^{I}(t)\sum _{j=1}^{12}{a}^{j}(t){b}^{j}(t){m}^{j}(t)+({\mu }_{H}+{\gamma }_{H}){I}_{H}^{I}(t)\\ \frac{d{R}_{H}^{I}(t)}{dt} & = & {\gamma }_{H}{I}_{H}^{I}(t)-{\mu }_{H}{R}_{H}^{I}(t)\\ \frac{d{S}_{M}^{I}}{dt} & = & -\frac{{S}_{M}^{I}(t)}{{N}_{H}^{I}}\sum _{j=1}^{12}{a}^{j}(t){c}^{j}(t){I}_{H}^{j}(t)+\sum _{j=1}^{12}{\mu }_{M}^{j}(t)[{L}_{M}^{I}(t)+{I}_{M}^{I}(t)]\\ \frac{d{L}_{M}^{I}}{dt} & = & \frac{{S}_{M}^{I}(t)}{{N}_{H}^{I}}\sum _{j=1}^{12}{a}^{j}(t){c}^{j}(t){I}_{H}^{j}(t)+\sum _{j=1}^{12}[{\mu }_{M}^{j}(t)+{\gamma }_{M}^{j}(t)]{L}_{M}^{I}(t)\\ \frac{d{I}_{M}^{I}}{dt} & = & \sum _{j=1}^{12}{\gamma }_{M}^{j}(t){L}_{M}^{I}(t)-\sum _{j=1}^{12}{\mu }_{M}^{j}(t){I}_{M}^{I}(t)\\ {N}_{H}^{I}(t) & = & {S}_{H}^{I}(t)+{I}_{H}^{I}(t)+{R}_{H}^{I}(t)\\ {N}_{M}^{I}(t) & = & {S}_{M}^{I}(t)+{L}_{M}^{I}(t)+{I}_{M}^{I}(t)\end{array}$$

Note that both the humans’ and mosquitoes’ populations are assumed to be constant and the last terms of the susceptible humans and susceptible mosquitoes are included to mimic births as equal to deaths. This is based on the assumption that dengue does not have any significant impact in humans nor mosquitoes in terms of mortality.

The values of the pulse-like parameters are given in^4^. For the sake of clarity, let us exemplify how the parameters in model (7) enter the code.Let us take the biting rate, for instance, *a*^*j*^(*t*). As shown in^4^, it varies with temperature and hence with time, that is, it varies seasonally. Its value should be written as *a*^*j*^(*t*) = *A*^*j*^[*θ*(*t* + *t*^*j*^) − *θ*(*t* − *t*^*j*+1^)], where *A*^*j*^ is the value of the biting rate obtained from^4^ for the month *j*. In other words, the biting rate assumes ‘discrete’ values in each month of the year.We did these calculations for Italy only where we used the average temperature month-by-month and applied the parameters from Table S2.

The densities ${I}_{H}^{I}(t)$ and ${I}_{M}^{I}(t)$ representing the autochthonous humans and mosquitoes infected by the infected travellers, ${I}_{H}^{I}(t)$, were obtained by numerically simulating model (7).

The above calculation is restricted to the autochthonous cases generated exclusively by infected travellers. If one is interested in the total number of dengue cases one should substitute the fourth and fifth equations of model (7) by:

7a $$\begin{array}{c}\frac{d{S}_{M}^{I}}{dt}=-\frac{{S}_{M}^{I}(t)}{{N}_{H}^{I}}\sum _{j=1}^{12}{a}^{j}(t){c}^{j}(t)[{I}_{H}^{j}(t)+{I}_{H}^{I}(t)]+\sum _{j=1}^{12}{\mu }_{M}^{j}(t)[{L}_{M}^{I}(t)+{I}_{M}^{I}(t)]\\ \frac{d{L}_{M}^{I}}{dt}=\frac{{S}_{M}^{I}(t)}{{N}_{H}^{I}}\sum _{j=1}^{12}{a}^{j}(t){c}^{j}(t)[{I}_{H}^{j}(t)+{I}_{H}^{I}(t)]+\sum _{j=1}^{12}[{\mu }_{M}^{j}(t)+{\gamma }_{M}^{j}(t)]{L}_{M}^{I}(t)\end{array}$$

## Results

**Figure 2:**
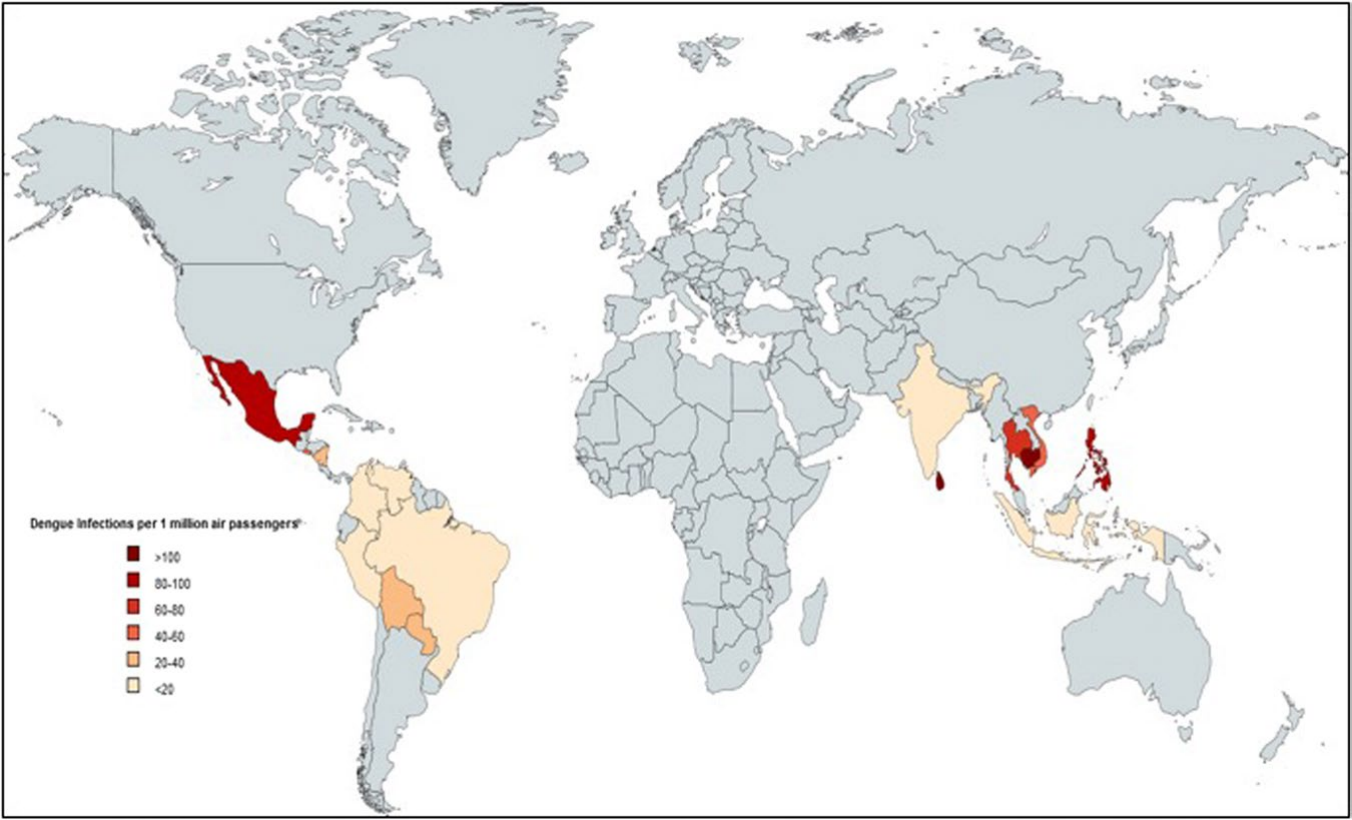
Selected countries with the highest reported number of dengue cases in 2012 (The values are normalized per 1 million passengers. Brazil has the highest absolute numbers but not per million**)**.

**Figure 3:**
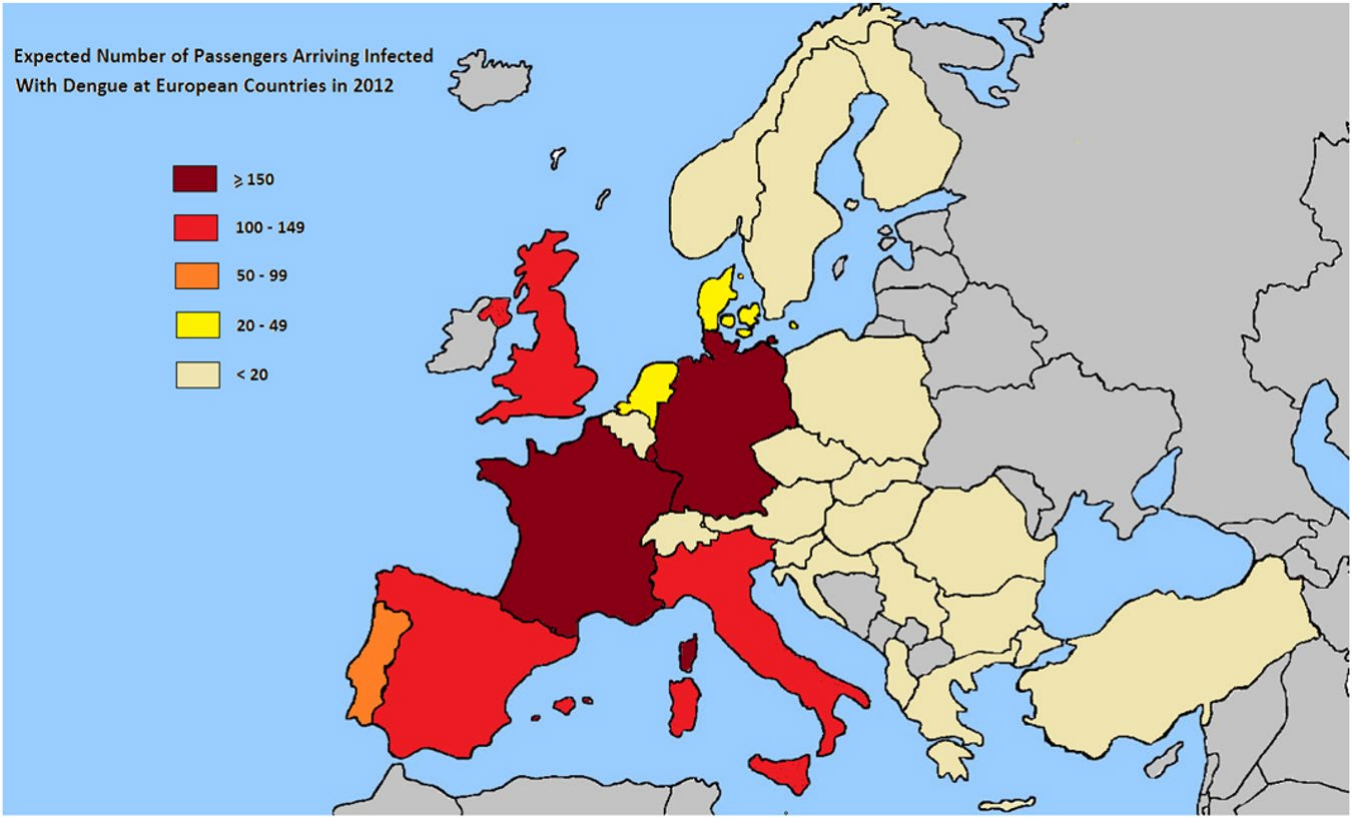
Expected number of dengue infections in air passengers arriving in European countries from the 16 selected countries in 2012.

**Figure 4:**
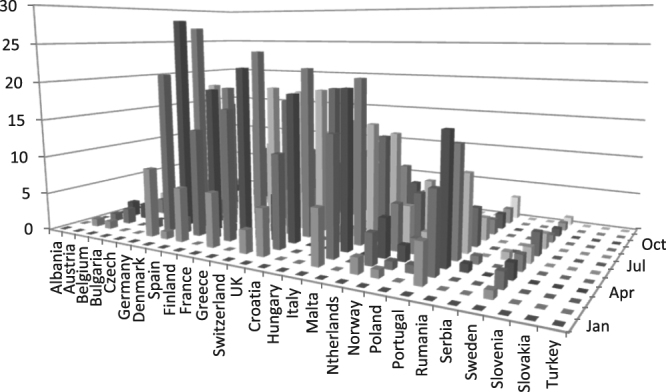
Expected Number of Passengers Arriving Infected With Dengue in Europe in 2012.

Figure 2 shows the countries that present more than 80% of the dengue burden (16 dengue endemic countries). Table 1 provides the probability of an air passenger being dengue viremia at the time of travel from the selected 16 exporting countries with the highest dengue incidence in the world to the 27 European destination countries for every month in the year 2012. Table 2 shows the expected number of dengue viremic air passengers from each of the 16 exporting countries, stratified by country of disembarkation and by country of arrival. Table 3 depicts the number of dengue viremic air passengers arriving in each of the 27 countries by month of arrival, and in total over the year of 2012. This table shows a range from zero to 167 dengue-viremic travelers arriving in each of the European importing countries. Germany receives the highest number of imported dengue-viremic air passengers (167), followed by France (150) and the United Kingdom (148). Figures 3 and 4 illustrate the countries that receive the highest amount of dengue virus importations via incoming air passengers.

**Table 1:** Probability of being infected with dengue (Prev. = equation (4)), number of travelers (Trav.), and expected number of infected people (Inf.) leaving the airports of the 16 selected countries by month in 2012.

Month	Brazil			Bolivia		
	Prev.	Trav.	Inf.	Prev.	Trav.	Inf.
Jan	4.55E-07	219536	0 ± 0	9.09E-06	10330	0 ± 0
Feb	1.75E-04	188345	33 ± 5	6.39E-05	8241	1 ± 0
Mar	3.35E-04	201075	67 ± 11	1.23E-04	6931	1 ± 0
Apr	4.40E-04	205969	91 ± 15	1.62E-04	5974	1 ± 0
May	4.00E-04	217757	87 ± 14	1.47E-04	8210	1 ± 0
Jun	2.57E-04	219886	57 ± 9	9.25E-05	5046	0 ± 0
Jul	1.26E-04	242269	31 ± 5	4.83E-05	4931	0 ± 0
Aug	6.18E-05	217028	13 ± 2	2.37E-05	6726	0 ± 0
Sep	4.66E-05	239181	11 ± 2	1.73E-05	6389	0 ± 0
Oct	5.11E-05	202519	10 ± 2	1.88E-05	4578	0 ± 0
Nov	6.11E-05	176584	11 ± 2	2.24E-05	3896	0 ± 0
Dec	7.28E-05	209612	15 ± 2	2.67E-05	4495	0 ± 0
Total		2539761	426 ± 68		75747	5 ± 1
	**El Salvador**			**Paraguay**		
Jan	1.56E-05	3193	0 ± 0	1.47E-05	4561	0 ± 0
Feb	4.30E-04	2267	1 ± 0	3.78E-04	3125	1 ± 0
Mar	8.26E-04	2599	2 ± 0	7.27E-04	3358	2 ± 0
Apr	1.09E-03	2236	2 ± 0	9.59E-04	3500	3 ± 0
May	9.91E-04	2300	2 ± 0	8.72E-04	2992	3 ± 0
Jun	6.22E-04	2257	1 ± 0	5.47E-04	2771	2 ± 0
Jul	3.26E-04	2838	1 ± 0	2.87E-04	2941	1 ± 0
Aug	1.60E-04	2965	0 ± 0	1.40E-04	3249	0 ± 0
Sep	1.17E-04	2810	0 ± 0	1.03E-04	2740	0 ± 0
Oct	1.26E-04	1979	0 ± 0	1.11E-04	2517	0 ± 0
Nov	1.51E-04	2178	0 ± 0	1.32E-04	2244	0 ± 0
Dec	1.79E-04	2312	0 ± 0	1.58E-04	2463	0 ± 0
Total		29934	12 ± 2		36461	14 ± 2
	**Thailand**			**Mexico**		
Jan	1.47E-06	329531	0 ± 0	8.33E-07	117193	0 ± 0
Feb	8.95E-06	321815	3 ± 0	1.07E-05	96836	1 ± 0
Mar	2.25E-05	343328	8 ± 0	2.69E-05	127676	3 ± 0
Apr	4.64E-05	287581	13 ± 1	5.55E-05	115295	6 ± 0
May	7.90E-05	198580	16 ± 1	9.46E-05	110254	10 ± 1
Jun	1.11E-04	159959	18 ± 1	1.33E-04	107635	14 ± 1
Jul	1.29E-04	204990	26 ± 2	1.54E-04	150928	23 ± 1
Aug	1.23E-04	263979	32 ± 2	1.47E-04	132677	20 ± 1
Sep	9.69E-05	177433	17 ± 1	1.16E-04	118734	14 ± 1
Oct	6.30E-05	190476	12 ± 0	7.55E-05	103697	8 ± 0
Nov	3.38E-05	232273	8 ± 0	4.05E-05	103867	4 ± 0
Dec	1.50E-05	255261	4 ± 0	1.80E-05	110462	2 ± 0
Total		2965206	157 ± 8		1395254	105 ± 5
	**VietNan**			**India**		
Jan	1.09E-06	37536	0 ± 0	8.33E-08	267443	0 ± 0
Feb	5.86E-06	45905	0 ± 0	3.17E-07	259617	0 ± 0
Mar	1.47E-05	56547	1 ± 0	8.04E-07	271524	0 ± 0
Apr	3.03E-05	49196	1 ± 0	1.67E-06	241751	0 ± 0
May	5.16E-05	38800	2 ± 0	2.87E-06	236148	1 ± 0
Jun	7.25E-05	32644	2 ± 0	4.05E-06	211554	1 ± 0
Jul	8.40E-05	41518	3 ± 0	4.70E-06	178268	1 ± 0
Aug	8.03E-05	60324	5 ± 0	4.49E-06	208148	1 ± 0
Sep	6.33E-05	37414	2 ± 0	3.53E-06	212047	1 ± 0
Oct	4.12E-05	37866	2 ± 0	2.29E-06	203889	0 ± 0
Nov	2.21E-05	44399	1 ± 0	1.22E-06	222536	0 ± 0
Dec	9.80E-06	42322	0 ± 0	5.36E-07	209710	0 ± 0
Total		524471	19 ± 1		2722635	6 ± 0
	**Colombia**			**Venezuela**		
Jan	2.05E-06	49522	0 ± 0	1.09E-06	37536	0 ± 0
Feb	6.68E-05	36188	2 ± 0	5.86E-06	45905	0 ± 0
Mar	1.28E-04	37645	5 ± 2	1.47E-05	56547	1 ± 0
Apr	1.69E-04	36004	6 ± 2	3.03E-05	49196	1 ± 0
May	1.54E-04	35016	5 ± 2	5.16E-05	38800	2 ± 0
Jun	9.66E-05	43833	4 ± 1	7.25E-05	32644	2 ± 0
Jul	5.05E-05	39760	2 ± 0	8.40E-05	41518	3 ± 0
Aug	2.47E-05	43313	1 ± 0	8.03E-05	60324	5 ± 2
Sep	1.81E-05	46471	1 ± 0	6.33E-05	37414	2 ± 0
Oct	1.96E-05	33870	1 ± 0	4.12E-05	37866	2 ± 0
Nov	2.34E-05	27996	1 ± 0	2.21E-05	44399	1 ± 0
Dec	2.79E-05	35964	1 ± 0	9.80E-06	42322	0 ± 0
Total		465582	29 ± 5		524471	19 ± 3
	**Nicaragua**			**Peru**		
Jan	1.82E-05	4319	0 ± 0	3.18E-06	37917	0 ± 0
Feb	3.84E-04	3103	1 ± 0	6.29E-05	32099	2 ± 0
Mar	6.99E-04	5559	4 ± 1	1.21E-04	35671	4 ± 1
Apr	9.06E-04	3354	3 ± 0	1.59E-04	34973	6 ± 2
May	8.40E-04	2138	2 ± 0	1.45E-04	35067	5 ± 2
Jun	5.59E-04	2373	1 ± 0	9.10E-05	32091	3 ± 0
Jul	2.67E-04	3203	1 ± 0	4.76E-05	36909	2 ± 0
Aug	9.15E-05	4725	0 ± 0	2.33E-05	47779	1 ± 0
Sep	2.25E-05	3055	0 ± 0	1.71E-05	42400	1 ± 0
Oct	3.99E-06	2294	0 ± 0	1.85E-05	35951	1 ± 0
Nov	5.10E-07	2437	0 ± 0	2.20E-05	31968	1 ± 0
Dec	4.70E-08	3628	0 ± 0	2.63E-05	31286	1 ± 0
Total		40188	12 ± 2		434111	26 ± 4
	**Philipines**			**Indonesia**		
Jan	9.62E-07	55280	0 ± 0	4.00E-07	39665	0 ± 0
Feb	8.37E-06	43699	0 ± 0	2.31E-06	35706	0 ± 0
Mar	2.43E-05	49190	1 ± 0	5.80E-06	48580	0 ± 0
Apr	5.58E-05	55224	3 ± 0	1.20E-05	53312	1 ± 0
May	1.01E-04	50788	5 ± 0	2.04E-05	57147	1 ± 0
Jun	1.46E-04	41218	6 ± 0	2.86E-05	60615	2 ± 0
Jul	1.73E-04	44283	8 ± 0	3.32E-05	58816	2 ± 0
Aug	1.60E-04	55526	9 ± 0	3.17E-05	93998	3 ± 0
Sep	1.20E-04	41590	5 ± 0	2.50E-05	66425	2 ± 0
Oct	7.65E-05	37065	3 ± 0	1.63E-05	62270	1 ± 0
Nov	4.63E-05	38321	2 ± 0	8.73E-06	50952	0 ± 0
Dec	3.24E-05	37204	1 ± 0	3.87E-06	47755	0 ± 0
Total		549388	43 ± 1		675241	12 ± 1
	**Sri Lanka**			**Cambodia**		
Jan	4.76E-06	39596	0 ± 0	6.45E-06	6809	0 ± 0
Feb	2.27E-05	43411	1 ± 0	1.81E-05	7247	0 ± 0
Mar	5.39E-05	44986	2 ± 0	5.25E-05	8484	0 ± 0
Apr	1.07E-04	33877	4 ± 0	1.21E-04	6269	1 ± 0
May	1.78E-04	32341	6 ± 0	2.20E-04	5695	1 ± 0
Jun	2.47E-04	20127	5 ± 0	3.16E-04	4332	1 ± 0
Jul	2.83E-04	29428	8 ± 0	3.75E-04	6739	3 ± 0
Aug	2.74E-04	46863	13 ± 0	3.46E-04	8259	3 ± 0
Sep	2.21E-04	39697	9 ± 0	2.60E-04	5710	1 ± 0
Oct	1.46E-04	35634	5 ± 0	1.66E-04	4193	1 ± 0
Nov	7.66E-05	30039	2 ± 0	1.00E-04	9208	1 ± 0
Dec	2.71E-05	27551	1 ± 0	7.01E-05	8508	1 ± 0
Total		229	56 ± 3			13 ± 1

**Table 2:** Expected number of dengue viremic air passengers from the 16 dengue endemic countries arriving in 27 European countries in 2012, by country of disembarkation.

	Brazil	Mexico	Philippines	Thailand	Indonesia	Viet Nam	India	Colombia
Albania	0	0	0	0	0	0	0	0
Austria	5	1	1	4	0	0	0	0
Belgium	5	2	1	6	0	0	0	0
Bulgaria	0	0	0	0	0	0	0	0
Czech	3	1	0	1	0	1	0	0
Germany	44	12	5	31	2	5	1	3
Dennmark	5	1	1	8	0	1	0	0
Spain	46	30	2	5	0	0	0	13
Finland	1	0	0	2	0	0	0	0
France	70	17	3	20	2	5	1	3
Greece	3	1	1	1	0	0	0	0
Switzerland	19	3	1	10	1	1	0	1
UK	50	13	13	32	2	3	2	3
Croatia	1	0	0	0	0	0	0	0
Hungary	2	0	0	1	0	0	0	0
Italy	69	13	6	10	1	1	1	3
Malta	0	0	0	0	0	0	0	0
Netherland	14	6	3	9	2	1	1	1
Norway	4	1	2	6	0	1	0	0
Poland	14	2	0	1	0	0	0	0
Portugal	68	1	0	1	0	0	0	0
Romania	1	0	0	1	0	0	0	0
Serbia	0	0	0	0	0	0	0	0
Sweden	4	1	1	8	0	0	0	0
Slovenia	0	0	0	0	0	0	0	0
Slovakia	0	0	0	0	0	0	0	0
Turkey	7	1	0	2	0	0	0	0
Total	426 ± 68	105 ± 5	43 ± 1	157 ± 8	12 ± 1	19 ± 1	6 ± 0	29 ± 5
	**Venezuela**	**Sri Lanka**	**Bolivia**	**El Salvador**	**Cambodia**	**Paraguay**	**Nicaragua**	**Peru**
Albania	0	0	0	0	0	0	0	0
Austria	0	1	0	0	0	0	0	0
Belgium	0	1	0	0	0	0	0	0
Bulgaria	0	0	0	0	0	0	0	0
Czech	1	1	0	0	0	0	0	0
Germany	5	8	0	1	1	6	2	2
Dennmark	1	1	0	0	0	0	0	0
Spain	0	0	3	4	0	3	4	7
Finland	0	0	0	0	0	0	0	0
France	5	5	1	2	8	1	2	5
Greece	0	0	0	0	0	0	0	0
Switzerland	1	4	0	0	0	1	0	1
UK	3	22	0	1	1	1	1	3
Croatia	0	0	0	0	0	0	0	0
Hungary	0	0	0	0	0	0	0	0
Italy	1	9	0	2	0	1	1	4
Malta	0	0	0	0	0	0	0	0
Netherland	1	1	0	0	1	0	1	1
Norway	1	0	0	0	0	0	0	1
Poland	0	0	0	0	0	0	0	0
Portugal	0	0	0	0	0	0	0	0
Romania	0	0	0	0	0	0	0	0
Serbia	0	0	0	0	0	0	0	0
Sweden	0	1	0	0	0	0	0	1
Slovenia	0	0	0	0	0	0	0	0
Slovakia	0	0	0	0	0	0	0	0
Turkey	0	0	0	0	0	0	0	0
Total	19 ± 3	56 ± 3	5 ± 1	12 ± 2	13 ± 1	14 ± 2	12 ± 2	26 ± 4

**Table 3:** Expected Number of Passengers Arriving Infected With Dengue at European Countries every month in 2012, and in total in the year 2012.

	Jan	Feb	Mar	Apr	May	Jun	Jul	Aug	Sep	Oct	Nov	Dec	Total
Albania	0	0	0	0	0	0	0	0	0	0	0	0	0 ± 1
Austria	0	1	1	2	2	2	2	2	1	1	1	0	15 ± 2
Belgium	0	1	2	2	2	2	3	3	1	1	1	0	18 ± 2
Bulgaria	0	0	0	0	1	0	0	0	0	0	0	0	1 ± 0
Czech	0	0	1	1	1	1	1	1	1	1	0	0	8 ± 1
Germany	0	9	21	28	27	19	16	16	12	8	6	5	167 ± 18
Denmark	0	1	2	2	3	2	4	3	1	1	1	0	20 ± 2
Spain	0	7	14	19	19	16	14	10	7	5	3	3	120 ± 13
Finland	0	0	1	1	1	1	1	0	0	0	0	0	5 ± 0
France	0	7	17	22	24	19	17	18	9	6	6	5	150 ± 16
Greece	0	0	1	1	1	1	1	1	0	0	0	0	6 ± 0
Switzerland	0	3	5	6	6	5	5	7	2	2	2	1	45 ± 5
UK	0	6	12	19	22	19	19	18	14	9	5	4	148 ± 16
Croatia	0	0	0	0	0	1	0	0	0	0	0	0	1 ± 0
Hungary	0	0	0	0	1	1	0	0	0	0	0	0	2 ± 0
Italy	0	7	15	20	21	15	13	13	8	5	3	3	124 ± 13
Malta	0	0	0	0	0	0	0	0	0	0	0	0	0 ± 0
Netherlands	0	2	4	5	6	5	6	7	3	2	1	1	42 ± 5
Norway	0	1	1	2	2	2	4	3	1	1	0	0	17 ± 2
Poland	0	0	1	1	1	1	1	0	0	0	0	0	5 ± 0
Portugal	0	5	10	16	14	10	5	3	2	2	2	3	71 ± 8
Rumania	0	0	0	1	1	0	0	0	0	0	0	0	2 ± 0
Serbia	0	0	0	0	0	1	0	0	0	0	0	0	1 ± 0
Sweden	0	1	2	2	2	2	3	2	1	1	1	1	18 ± 2
Slovenia	0	0	0	0	0	0	0	0	0	0	0	0	0 ± 0
Slovakia	0	0	0	0	0	0	0	0	0	0	0	0	0 ± 0
Turkey	0	0	0	0	0	0	0	0	0	0	0	0	0 ± 0

### Autochthonous transmission as a result of dengue virus importation

The risk of onward transmission via *Aedes* mosquitoes depends on the presence of such mosquitoes, and Fig. 1 shows the *Aedes albopictus* distribution in Europe. From Fig. 1 it is evident that only Italy has nationwide presence of *Aedes albopictus*. As the air passenger flight information was available only at a country level, we were only able to calculate the probability of onward transmission where the presence of *Aedes albopictus* is countrywide. Hence, we selected Italy to illustrate the method to calculate the number of secondary autochthonous cases in one year as a result of dengue virus importation via incoming air passengers from dengue endemic countries. Table 3 shows the monthly number of dengue viremic air passengers arriving in Italy. Of the total number of 124 infected travellers to Italy in one year, 51 arrived in the summer months of June, July and August, a seasonal window with the most suitable vectorial capacity for *Aedes* mosquitoes based on previous calculations on the seasonal variation of vectorial capacity in different European cities by Liu-Helmersson *et al*.^4^. Out model estimated that 10autochthonous (secondary) cases would occur as a result of 51 air passengers arriving in Italy at a time of being dengue viremic. These 10 persons include 2 (20%) symptomatic dengue cases, assuming a 4:1 ratio of asymptomatic to symptomatic infections.

We also validated our models against the well-documented dengue outbreak in Madeira with 2180 reported dengue cases. We estimated the risk of autochthonous transmission for the Madeira Islands where *Aedes aegypti* was responsible for a large dengue outbreak in 2012, as well as the expected number of secondary cases resulting from the introduction of imported cases from Venezuela and Brazil. Our models estimated the per capita probability to be 0.035 and the expected number of secondary cases to be 2205, which is well in agreement with the 2180 reported cases in the 2012 outbreak.

Table 4 summarizes the cumulative per capita risk and expected number of autochthonous cases for Italy (*Aedes albopictus*) and the Madeira Islands (*Aedes aegypti*). The parameters used for the simulations can be found in Table S2 (*Aedes albopictus*) and S3 (*Aedes aegypti*) of the supplementary material.

**Table 4:** Cumulative per capita risk and expected number of autochthonous cases for Italy(*Aedesalbopictus*) and the Madeira Islands (*Aedesaegypti*). Parameters used for the simulations as in Table S2 (*Aedesalbopictus*) and S3 (*Aedesaegypti*) of the supplementary material.

	Per capita risk	Expected number of autochthonouscases
Italy	1.67E-7	10 ± 1
Madeira	3.35E-2	2205 ± 236

## Discussion

Although reported autochthonous cases in Europe in the recent past highlight the threat of dengue to Europe^27^, the extent of such a threat has not been quantified. This is the first attempt to quantify the actual number of dengue viremic air passengers from dengue endemic countries into Europe -an important parameter that determines the risk calculations of subsequent local dengue virus outbreaks. As not only clinically apparent viremic cases transmit dengue viruses^28^, we also included asymptomatic infections into our calculations at a published ratio of 4:1. Our estimated number of importations tally with the reported importations of dengue in Europe, although the numbers are slightly lower as we are focusing only on air passengers traveling to Europe still in the viremic phase (e.g. persons traveling at a time of viremia for the short period within 5 days after onset of symptoms)^20,29^. The majority of dengue cases reported in returning travelers to Europe are not viremic anymore at the time of arrival^20^.

The highest number of dengue virus importations via air travelers were modeled to occur in Germany, France and the United Kingdom. *Aedes albopictus* was recently introduced to Kent, United Kingdom but its distribution is very limited and not yet established^30^. In Germany, many more *Aedes albopictus* have been reported and established populations have been recorded in parts of Southern Germany^2,31^. However, risk for local transmission is limited under current climatic conditions. Both France and Italy receive a significant number of modeled dengue infected air passengers and both countries have significant presence of *Aedes albopictus*- indeed France experienced autochthonous dengue transmission in the year 2010 in Nice in the Southern part of its country^7^ and Italy experienced a chikungunya outbreak in 2007 in the Northern part of Italy^32^. We were limited to passenger flight information at a country level, so we restricted our analysis of the risk of onward transmission of dengue to Italy, a country where *Aedes albopictus* is distributed across the majority of the country.

For Italy, we modeled the probability for an imported case to result in secondary cases by taking into account the monthly vectorial capacity where transmission is most suitable (eg the summer months of June, July and August)^4^. We translated these findings into per capita probability in Italy. The per capita probability was as low as 0.000000167, and we estimated 10 secondary infections which include around 2 symptomatic cases. For Madeira, we modeled the probability of an imported case to result in secondary cases to be 0.035 and the expected number of secondary cases to be 2205. The difference in the mosquitoes’ species between Italy and Madeira is crucial in appreciating the risk difference between the two countries/areas. As *Aedes albopictus* is a much less competent mosquito for dengue transmission than *Aedes aegypti*, a large number of imported infections to Italy resulted in a small number of autochthonous cases, whereas a very small number of imported infections to Madeira resulted in a major outbreak.

Our models have several limitations: From an empirical point of view, there is a need for accurate data on dengue incidence in the origin countries. We relied on notified dengue cases to WHO, which is probably a significant underestimate as recent modeling estimates showed at least 10 to 100 fold higher numbers^33^. Another limitation is the timing of imports. Here we were restricted to two countries as reference points for fitting monthly incidence curves. However, the highest uncertainties in our model were related to mosquito densities in Europe. There are no published data on the *Aede s*mosquito abundance in relation to the host population which is a crucial parameter for modelling, nor is there sufficient information on biting rates, extrinsic incubation period, mosquito longevity under temperate climate conditions except for some pivotal temperature driven data derived from the work by Louis Lambrechts’ team at the Institute Pasteur^34,35^.

We selected the year 2012 for our model parameters (dengue incidence in originating countries and air travel volume) in order to validate our model against the actual dengue outbreak that took place in Madeira in that year. Validating our model against the reported dengue outbreak in Madeira in 2012, we found that our model fits very well with the true number of cases reported: our model results estimated 2205 autochthonous infectious, which is well in agreement with the 2180 reported cases in the 2012 outbreak. Therefore, despite its limitations, the model proposed here can be very useful in the understanding of the risk of dengue virus importation into still unaffected areas, and calculate the probability of secondary cases in those areas where susceptible *Aedes* mosquitoes exist. This paper is hence intended mainly as a novel methodological proposal to estimate the risk of dengue introductions into Europe and subsequent probability and numbers of secondary (eg locally transmitted) infections.

In conclusion, our estimates highlight that the risk is overall low which probably represents a good approximation of reality.

## Electronic supplementary material

Supplementary Material

## References

[1] Wilder-Smith A (2017). Epidemic arboviral diseases: priorities for research and public health. Lancet Infect Dis.

[2] Kraemer MU (2015). The global distribution of the arbovirus vectors Aedes aegypti and Ae. albopictus. Elife.

[3] Reiter P (1998). Aedes albopictus and the world trade in used tires, 1988–1995: the shape of things to come?. J Am Mosq Control Assoc.

[4] Liu-Helmersson J (2016). Climate Change and Aedes Vectors: 21st Century Projections for Dengue Transmission inEurope. EbioMedicine.

[5] Liu-Helmersson J, Stenlund H, Wilder-Smith A, Rocklov J (2014). Vectorial Capacity of Aedes aegypti: Effects of Temperature and Implications for Global Dengue Epidemic Potential. PLoS One.

[6] Astrom C (2012). Potential distribution of dengue fever under scenarios of climate change and economic development. Ecohealth.

[7] La Ruche G (2010). First two autochthonous dengue virus infections in metropolitan France, September 2010. Euro Surveill.

[8] Wilder-Smith A (2014). The 2012 dengue outbreak in Madeira: exploring the origins. Euro Surveill.

[9] Khan, K. *et al*. Assessing the origin of and potential for international spread of chikungunya virus from the Caribbean. *PLoS currents* **6**, 10.1371/currents.outbreaks.2134a0a7bf37fd8d388181539fea2da5 (2014). 10.1371/currents.outbreaks.2134a0a7bf37fd8d388181539fea2da5 PMC4055609 24944846

[10] Khan, K. *et al*. Potential for the international spread of middle East respiratory syndrome in association with mass gatherings in saudi arabia. *PLoS currents* **5**, 10.1371/currents.outbreaks.a7b70897ac2fa4f79b59f90d24c860b8 (2013). 10.1371/currents.outbreaks.a7b70897ac2fa4f79b59f90d24c860b8 PMC3714242 23884087

[11] Khan K (2009). Spread of a novel influenza A (H1N1) virus via global airline transportation. N Engl J Med.

[12] Quam, M. B. & Wilder-Smith, A. Estimated global exportations of Zika virus infections via travellers from Brazil from 2014 to 2015. *J Travel Med* **23**, 10.1093/jtm/taw059 (2016). 10.1093/jtm/taw059 27601533

[13] Quam MB, Sessions O, Kamaraj US, Rocklov J, Wilder-Smith A (2016). Dissecting Japan’s Dengue Outbreak in 2014. Am J Trop Med Hyg.

[14] Massad E, Tan SH, Khan K, Wilder-Smith A (2016). Estimated Zika virus importations to Europe by travellers from Brazil. Glob Health Action.

[15] Hamer, D. H. *et al*. Travel-Associated Zika Virus Disease Acquired in the Americas Through February 2016: A GeoSentinel Analysis. *Ann Intern Med*, 10.7326/M16-1842 (2016). 10.7326/M16-1842 27893080

[16] Burattini MN (2016). Potential exposure to Zika virus for foreign tourists during the 2016 Carnival and Olympic Games in Rio de Janeiro, Brazil. Epidemiol Infect.

[17] Wilder-Smith A (2015). Potential for international spread of wild poliovirus via travelers. BMC Med1.

[18] Zhou YP, Wilder-Smith A, Hsu LY (2014). The role of international travel in the spread of methicillin-resistant Staphylococcus aureus. J Travel Med.

[19] Glaesser, D., Kester, J., Paulose, H., Alizadeh, A. & Valentin, B. Global travel patterns: an overview. *J Travel Med* **24**10.1093/jtm/tax007 (2017). 10.1093/jtm/tax007 28637267

[20] Neumayr, A. *et al*. Sentinel surveillance of imported dengue via travellers toEurope 2012 to 2014: TropNet data from the DengueTools Research Initiative. E*uro Surveill* **22**10.2807/1560-7917.ES.2017.22.1.30433 (2017). 10.2807/1560-7917.ES.2017.22.1.30433 PMC5388098 28080959

[21] Lopez LF (2016). Modeling Importations and Exportations of Infectious Diseases via Travelers. Bull Math Biol.

[22] Quam MB, Wilder-Smith A (2015). Importation index of dengue to determine the most probable origin of importation. J Travel Med.

[23] Quam MB (2015). Estimating air travel-associated importations of dengue virus into Italy. J Travel Med.

[24] Ximenes R (2016). The risk of dengue for non-immune foreign visitors to the 2016 summer olympic games in Rio de Janeiro, Brazil. BMC Infect Dis.

[25] Massad E (2014). Risk of symptomatic dengue for foreign visitors to the 2014 FIFA World Cup in Brazil. Mem Inst Oswaldo Cruz.

[26] Amaku M (2014). A comparative analysis of the relative efficacy of vector-control strategies against dengue fever. Bull Math Biol.

[27] Schaffner F, Fontenille D, Mathis A (2014). Autochthonous dengue emphasises the threat of arbovirosis in Europe. Lancet Infect Dis.

[28] Duong V (2015). Asymptomatic humans transmit dengue virus to mosquitoes. Proc Natl Acad Sci USA.

[29] Semenza JC (2014). International dispersal of dengue through air travel: importation risk forEurope. PLoS Negl Trop Dis.

[30] Medlock JM (2017). Detection of the invasive mosquito species Aedes albopictus in southern England. Lancet Infect Dis.

[31] Kraemer, M. U. *et al*. The global compendium of Aedes aegypti and Ae. albopictus occurrence. *Scientific data* **2**, 150035, 10.1038/sdata.2015.35 (2015). 10.1038/sdata.2015.35 PMC4493829 26175912

[32] Rezza G (2007). Infection with chikungunya virus in Italy: an outbreak in a temperate region. Lancet.

[33] Stanaway JD (2016). The global burden of dengue: an analysis from the Global Burden of Disease Study 2013. Lancet Infect Dis.

[34] Carrington LB, Seifert SN, Willits NH, Lambrechts L, Scott TW (2013). Large diurnal temperature fluctuations negatively influence Aedes aegypti (Diptera: Culicidae) life-history traits. Journal of medical entomology.

[35] Lambrechts L (2011). Impact of daily temperature fluctuations on dengue virus transmission by Aedes aegypti. Proc Natl Acad Sci USA.

