# Supplementary material for: Estimating the probability of dengue virus introduction and secondary autochthonous cases in Europe
Source: Sci Rep. 2018 Mar 15;8:4629. doi: 10.1038/s41598-018-22590-5 (PMC5854675; doi:10.1038/s41598-018-22590-5)
Supplement: Supplementary file 1 — Supplementary Material [file 41598_2018_22590_MOESM1_ESM.docx]

**Estimating the probability of dengue virus introduction and secondary autochthonous cases in Europe**

***Eduardo Massad^1,2,3,4^, Marcos Amaku^1^, Francisco Antonio Bezerra Coutinho^1^, Claudio José Struchiner^5^, Marcelo Nascimento Burattini^1,6^***, ***Kamran Khan^7^, Jing Liu-Helmersson^8^, Joacim Rocklöv^,8^ Moritz U.G. Kraemer^9^andAnnelies Wilder-Smith^8,10,11^***

^1^School of Medicine, University of Sao Paulo, Brazil

^2^London School of Hygiene and Tropical Medicine, UK

^3^College of Natural and Life Sciences, The University of Derby, UK

^4^ School of Applied Mathematics, FundaçãoGetúlio Vargas, Rio de Janeiro, Brazil

^5^Programme ofScientificComputation,Fundação Oswaldo Cruz, Rio de Janeiro, Brazil

^6^Hospital São Paulo, Escola Paulista de Medicina, Universidade Federal de São Paulo, São Paulo, SP, Brazil

^7^Li KaShing Knowledge Institute, St Michael’s Hospital, Toronto, Canada

^8^Department Public Health and Clinical Medicine, Epidemiology and Global Health, Umea° University, SE-901 85 Umea°, Sweden;

^9^Harvard Medical School & Boston Children's Hospital & Department of Zoology, University of Oxford, UK;

^10^Institute of Public Health, University of Heidelberg, Germany;

^11^Lee Kong Chian School of Medicine, Nanyang Technological University, Singapore.

**Supplementary Material**

| Table S1. Countries which reported 95% of dengue cases in 2012. | | |
| --- | --- | --- |
| Country | **Reported Cases** | **Percentage of World cases** |
| Brazil | 565510 | 34 % |
| Mexico | 164947 | 10 % |
| Philippines | 154945 | 9 % |
| Thailand | 78063 | 5 % |
| Indonesia | 74062 | 4 % |
| Viet Nam | 69023 | 4 % |
| India | 50222 | 3 % |
| Colombia | 49361 | 3 % |
| Venezuela | 49044 | 3 % |
| Sri Lanka | 44456 | 3 % |
| Bolivia | 42704 | 3 % |
| El Salvador | 41793 | 2 % |
| Cambodia | 40164 | 2 % |
| Paraguay | 39063 | 2 % |
| Nicaragua | 30499 | 2 % |
| Peru | 29994 | 2 % |
| Total | 1523850 | 91 % |

| Table S2. Parameters used to calculate the risk for Italy in which *Aedes albopictus* is present (from reference [5]). | | | | | | | |
| --- | --- | --- | --- | --- | --- | --- | --- |
| Month | T (^o^C)* | γ_M_  _Inverse of the extrinsic incubation period_ | μ_M_  _Mosquitoes' natural mortality rate_ | *a*  _biting rate_ | *N_M_/N_H_*  _Mosquitoes' density with respect to humans_ | *c*  _Probability of transmission to mosqutioes_ | *b*  _Probability of transmission to humans_ |
| Jan | 14 | 0.0287 | 0.024 | 0.07 | 0.94 | 0.12 | 0.11 |
| Feb | 12 | 0.0230 | 0.023 | 0.1 | 0.67 | 0 | 0 |
| Mar | 15 | 0.0287 | 0.024 | 0.07 | 0.94 | 0.12 | 0.11 |
| Apr | 17 | 0.0453 | 0.027 | 0.07 | 1.09 | 0.26 | 0.25 |
| May | 19 | 0.0438 | 0.031 | 0.08 | 1.10 | 0.41 | 0.41 |
| Jun | 26 | 0.0906 | 0.032 | 0.21 | 1.08 | 0.99 | 0.66 |
| Jul | 28 | 0.1065 | 0.031 | 0.23 | 1.15 | 1 | 0.68 |
| Aug | 29 | 0.10092 | 0.029 | 0.25 | 1.16 | 1 | 0.65 |
| Sep | 25 | 0.0835 | 0.033 | 0.2 | 1.05 | 0.92 | 0.64 |
| Oct | 22 | 0.1065 | 0.034 | 0.14 | 1.01 | 0.7 | 0.5 |
| Nov | 18 | 0.0438 | 0.031 | 0.08 | 1.10 | 0.41 | 0.29 |
| Dec | 14 | 0.0287 | 0.024 | 0.07 | 0.94 | 0.12 | 0.08 |

*Source: *http://www.esrl.noaa.gov/psd/*

| Table S3. Parameters used to calculate the risk (from[5]) for Madeira in which *Aedes aegyptis* is present. | | | | | | | |
| --- | --- | --- | --- | --- | --- | --- | --- |
| Month | T (^o^C)* | γ_M_  _Inverse of the extrinsic incubation period_ | μ_M_  _Mosquitoes' natural mortality rate_ | *a*  _biting rate_ | *N_M_/N_H_*  _Mosquitoes' density with respect to humans_ | *c*  _Probability of transmission to mosqutioes_ | *b*  _Probability of transmission to humans_ |
| Jan | 16 | 0.0287 | 0.027 | 0.07 | 1.27 | 0.26 | 0.18 |
| Feb | 16 | 0.0287 | 0.027 | 0.07 | 1.27 | 0.26 | 0.18 |
| Mar | 16 | 0.0287 | 0.027 | 0.07 | 1.27 | 0.26 | 0.18 |
| Apr | 17 | 0.0453 | 0.031 | 0.08 | 1.13 | 0.41 | 0.29 |
| May | 18 | 0.0438 | 0.031 | 0.08 | 1.13 | 0.41 | 0.29 |
| Jun | 20 | 0.0906 | 0.033 | 0.11 | 1.04 | 0.55 | 0.40 |
| Jul | 22 | 0.1065 | 0.034 | 0.14 | 1.01 | 0.70 | 0.50 |
| Aug | 23 | 0.0835 | 0.033 | 0.18 | 1.03 | 0.85 | 0.60 |
| Sep | 23 | 0.0835 | 0.033 | 0.18 | 1.03 | 0.85 | 0.60 |
| Oct | 21 | 0.0906 | 0.033 | 0.11 | 1.04 | 0.55 | 0.40 |
| Nov | 19 | 0.0438 | 0.031 | 0.08 | 1.13 | 0.41 | 0.29 |
| Dec | 17 | 0.0453 | 0.031 | 0.08 | 1.13 | 0.41 | 0.29 |

*Source: *https://pt.climate-data.org/location/144/*

**Figure S1. Seasonal distribution of reported dengue cases in Brazil in 2012 (**From SINAN, Minitry of Health of Brazil.**).**

**Figure S2. Seasonal distribution of reported dengue cases in Thailand in 2012 (**from WHO**).**

Figures S3 and S4 show the quality of fitting of equation (2) to real data for Brazil and Thailand, respectively

**Figure S3. Fitting quality of equation (2) (line) to the officially reported number of dengue cases (dots) multiplied by 4 (total infections) for Brazil in 2012 (RMS-error=28087.3).**

**Figure S4. Fitting quality of equation (2) (line) to the officially reported number of dengue cases (dots) multiplied by 4 (total infections) for Thailand in 2012 (RMS-error=3438.36).**
